# Catheter ablation of atrial fibrillation in patients with autoimmune disease: A propensity score matching study based on the China Atrial Fibrillation Registry

**DOI:** 10.1002/clc.24036

**Published:** 2023-05-22

**Authors:** Ming‐Yang Gao, Li‐Hong Huang, Yi‐Wei Lai, Qi Guo, Xue‐Yuan Guo, Song‐Nan Li, Chen‐Xi Jiang, Nian Liu, Liu He, Xu Li, Ri‐Bo Tang, Xin Du, De‐Yong Long, Cai‐Hua Sang, Jian‐Zeng Dong, Chang‐Sheng Ma

**Affiliations:** ^1^ Department of Cardiology, National Clinical Research Center for Cardiovascular Diseases, Beijing Anzhen Hospital Capital Medical University Beijing China

**Keywords:** atrial fibrillation, autoimmune disease, catheter ablation, recurrence

## Abstract

**Background:**

Evidence on outcomes of catheter ablation (CA) for atrial fibrillation (AF) in patients with autoimmune disease (AD) is limited.

**Hypothesis:**

Patients with AD had worse outcomes after CA procedures for AF.

**Methods:**

A retrospective analysis was performed in patients undergoing AF ablation between 2012 and 2021. The risk of recurrence after ablation was investigated in patients with AD and a 1:4 propensity score matched non‐AD group.

**Results:**

We identified 107 patients with AD (64 ± 10 years, female 48.6%) who were matched with 428 non‐AD patients (65 ± 10 years, female 43.9%). Patients with AD exhibited more severe AF‐related symptoms. During the index procedure, a higher proportion of AD patients received nonpulmonary vein trigger ablation (18.7% vs. 8.4%, *p* = 0.002). Over a median follow‐up of 36.3 months, patients with AD experienced a similar risk of recurrence with the non‐AD group (41.1% vs. 36.2%, *p* = 0.21, hazard ratio [HR]: 1.23, 95% confidence interval [CI]: 0.86–1.76) despite a higher incidence of early recurrences (36.4% vs. 13.5%, *p* = 0.001). Compared with non‐AD patients, patients with connective tissue disease were at an increased risk of recurrence (46.3% vs. 36.2%, *p* = 0.049, HR: 1.43, 95% CI: 1.00–2.05). Multivariate Cox regression analysis showed that the duration of AF history and corticosteroid therapy were independent predictors of postablation recurrence in patients with AD.

**Conclusions:**

In patients with AD, the risk of recurrence after ablation for AF during the follow‐up was comparable with non‐AD patients, but a higher risk of early recurrence was observed. Further research into the impact of AD on AF treatment is warranted.

## Graphical abstract

In this study conducted at Beijing Anzhen Hospital, we investigated the association between autoimmune diseases (AD) and procedural characteristics and recurrence after catheter ablation for atrial fibrillation in 107 patients with AD (AD group) and 428 propensity score‐matched patients without AD (non‐AD group). During the index procedure, a higher proportion of AD patients received nonpulmonary vein trigger ablation. Over a median follow‐up of 36.3 months, patients with AD experienced a similar risk of recurrence with the non‐AD group despite a higher incidence of early recurrences.

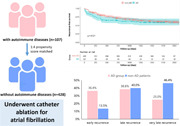

## 1 INTRODUCTION

Previous research had revealed that selected autoimmune diseases (AD), such as rheumatoid arthritis (RA) and systemic lupus erythematosus (SLE), are associated with an elevated risk of developing cardiovascular disease.^1^, ^2^, ^3^ However, the impact of AD on the incidence and outcome of arrhythmic diseases is less explored. Recently, a large‐scale population‐based study revealed that the hazard ratio (HR) of developing atrial fibrillation (AF) or atrial flutter was 1.40 (95% confidence interval [CI]: 1.35–1.46) in AD patients compared to the general population.^1^ Other sporadic evidence also showed that the risk of developing AF is higher in patients with RA, ankylosing spondylitis (AS), and psoriasis than in the general population.^4^, ^5^, ^6^, ^7^ This risk is elevated through multifaceted mechanisms. Beyond a higher prevalence of conventional risk factors,^4^ autoimmunity also contributes to the occurrence of AF in a complicated way, including direct effects on connective tissue and small vessels, cardiomyocytes, and possibly from some AD treatments. Systemic inflammatory states can evoke a proinflammatory transformation in the epicardial adipose tissue, resulting in the release of proinflammatory adipocytokines^8^, ^9^ and myocardial fibrosis in the adjacent atrium. Eventually, atrial myopathy may be developed and lead to increased stiffness of the atrial wall and reduced mechanical atrial functions.

Catheter ablation (CA) has recently become a mainstream AF treatment.^10^ However, recurrences of AF after the ablation procedure are relatively common and are influenced by factors including inflammation status, atrial fibrosis, and so forth. A recent survey showed that CA for tachyarrhythmia is often withheld in patients with AD at the physician's discretion for fear of the high risk of procedure‐related complications and recurrences.^11^ Up to now, the safety and efficacy of CA for AF in patients with AD have not been evaluated. In this research, we evaluated the outcome of CA for AF in patients with AD.

## 2 METHODS

### 2.1 Study design and study population

Patients who underwent CA for AF with a confirmed diagnosis of AD at Beijing Anzhen Hospital were retrospectively screened from the China Atrial Fibrillation Registry, which is a prospective registry study launched in 2011 (Chinese Clinical Trial Registry ChiCTR‐OCH‐13003729).^12^ Patients with previous ablation history for AF, valvular disease, previous cardiac surgery, congenital heart disease, cardiomyopathy, advanced heart failure (HF) (New York Heart Association Class III–IV), and who were diagnosed with AD after the CA procedure were excluded. A propensity score (PS) matching approach was performed to match patients without AD (the non‐AD group) and the AD group at a ratio of 4:1. Potential confounding factors including age, gender, AF type, duration of AF history, and comorbidities (stroke, HF, hypertension [HTN], coronary artery disease [CAD], peripheral artery disease, hyperlipidemia, chronic kidney disease [CKD], and diabetes mellitus [DM]) was fit into a multivariable logistic model with a caliper as 0.1.

The primary outcome of this study was the postablation recurrences, which were further classified into early recurrence (within the first 3 months), late occurrence (between 3 months and 1 year), and very late recurrence (beyond 1 year). Characteristics of CA procedures and periprocedural adverse events were documented and analyzed. All outcomes are compared in patients with and without AD, and in subgroups of AD patients including (1) with connective tissue disease (CTD, such as RA, AS, SLE) or only had organ‐specific diseases (such as Hashimoto thyroiditis, psoriasis) based on the location where autoimmunity attack happens^1^; (2) with single or multiple AD.

### 2.2 Data collection

Baseline characteristics, including gender, age, AF type and duration, the severity of AF‐related symptoms (European Heart Rhythm Association score [EHRA]), comorbidities, as well as the echocardiographic index including left atrial diameter (LAD), left ventricular ejection fraction (LVEF), systolic and mean pulmonary artery pressure (sPAP and mPAP) were collected. Levels of hypersensitive C‐reactive protein (hs‐CRP), white blood cell, and differential leukocyte counts were documented. Regular medical therapy (restricted to corticosteroids and disease‐modifying antirheumatic drugs [DMARDs]) for patients with AD was recorded.

### 2.3 CA procedure

General principles of ablation protocol at our center have been reported.^13^, ^14^ Three‐dimensional electroanatomical mapping was performed with the CARTO 3 system (Biosense Webster) and ablation was performed with a standard 3.5 mm irrigated ablation catheter. Routine ablation strategy included pulmonary vein isolation (PVI) for paroxysmal atrial fibrillation (PAF) and a “2C3L” protocol for persistent atrial fibrillation (PsAF)^13^ (PVI and linear ablation at LA roofline [RL], mitral isthmus [MI], and cavotricuspid isthmus [CTI]). Complex fractionated atrial electrograms (CFAE) ablation was also allowed at the operators' discretion. For patients with PsAF, if AF was not terminated upon completing the 2C3L protocol, cardioversion would be performed to restore sinus rhythm. Under sinus rhythm, PVI and the linear block would be verified. Detection and elimination of non‐PV triggers would be performed by: (1) focusing on the first beat triggering AF; (2) programmed pacing from the right atrium with a cycle length of 200–300 ms for 10 seconds; (3) high dose isoproterenol infusion (20–30 mg/min for 10–15 minutes). If triggering activities appeared spontaneously or by induction measures, mapping and ablation would be carried out. For PAF patients, if PVI and non‐PV triggers elimination were completed but sustained AF could still be induced, extra linear ablation would be carried out at the operator's discretion. The endpoint of the ablation procedure was PVI, elimination of non‐PV triggers, and complete linear block if conducted.

### 2.4 Follow‐up

Twelve‐lead electrocardiogram(ECG) and transthoracic echocardiography were routinely performed the day after the procedure. Twenty‐four‐hour dynamic ECG at 1, 2, 3, and 6 months after the procedure, as well as incident ECG during symptom onset were reviewed for any arrhythmia recurrence (defined as AF/atrial tachycardia >30 seconds). Outpatient revisits and telephone follow‐up was carried out to collect information on postprocedural adverse events and redo procedures. All patients without contraindications were described with propafenone or amiodarone for 3 months, which would be discontinued if the patients presented with stable sinus rhythm.

### 2.5 Statistical analysis

Baseline characteristics were presented as mean (standard deviation) or median (interquartile range). The Student *t* test or Mann–Whitney *U* test was applied for between‐group comparisons. Categorical statistics are analyzed by the Kruskal–Wallis test. Dichotomous variables are presented as numbers and percentages and were compared by *χ* ^2^ test or Fisher Exact test. Survival analysis was visualized by Kaplan–Meier curves and significance tests between groups were conducted by the log‐rank test. Univariate and multivariate Cox regression models were constructed to investigate the relationship between potential covariates and outcomes. Proportional hazard assumptions were checked by the Schoenfeld residuals test. Results are presented as HRs with 95% CIs. Two‐sided *p* < 0.05 was considered statistically significant. The statistical analysis was performed using R version 4.2.1.

## 3 RESULTS

### 3.1 Baseline characteristics

Between 2012 and 2021, we identified 107 patients with AD fulfilling the inclusion criteria (mean age 64 ± 10 years, female 48.6%, PsAF 34.6%). These patients constituted the AD group and were further divided into subgroups including (1) CTD group (*n* = 82) and organ‐specific disease group (*n* = 25) and; (2) single AD (*n* = 92) and multiple ADs (*n* = 15). RA is the most common AD (*n* = 41), followed by AS (*n* = 23), Hashimoto thyroiditis (*n* = 17), Sjogren‘s Syndrome (*n* = 15), psoriasis (*n* = 13), autoimmune hepatitis (*n* = 5), Grave disease (*n* = 4), multiple arteritis (*n* = 1), SLE (*n* = 1), systemic sclerosis (*n* = 1), and polymyalgia rheumatica (*n* = 1).

After PS matching, 428 AF patients (mean age 65 ± 10 years, female 43.9%, PsAF 36.7%) without AD were identified and constituted the non‐AD group. Table 1 summarizes the baseline characteristics of AD and non‐AD groups. Patients with AD had a significantly higher grade of EHRA score (*p* < 0.001). In addition, patients with AD presented with higher sPAP (32.6 ± 7.3 vs. 30.3 ± 7.5 mmHg, *p* = 0.011) and mPAP (22.0 ± 4.8 vs. 20.5 ± 5.1 mmHg, *p* = 0.009).

**Table 1:** Baseline characteristics.

Variable	AD (*n* = 107)	Non‐AD (*n* = 428)	*P*1	Connective tissue diseases (*n* = 82)	Organ‐specific diseases (*n* = 25)	*P*2	Single AD (*n* = 92)	Multiple AD (*n* = 15)	*P*3
Age, years	64 ± 10	65 ± 10	0.721	65 ± 10	61 ± 9	0.092	64 ± 10	69 ± 8	0.060
Female, *n* (%)	52 (48.6)	188 (43.9)	0.385	42 (51.2)	10 (40.0)	0.326	39 (42.4)	13 (86.7)	0.001
Persistent, *n* (%)	37 (34.6)	157 (36.7)	0.686	28 (34.1)	9 (36.0)	0.865	34 (37.0)	3 (20.0)	0.162
Median AF duration, year (IQR)	2.0 (0.75, 5.0)	2.3 (0.6, 5.3)	0.788	2.0 (0.5, 5.0)	3.0 (1.0, 4.0)	0.507	2.0 (0.6, 5.0)	2.5 (0.8, 6.0)	0.907
Median AD duration, year (IQR)	–	–	–	10.0 (5.0, 20.0)	10.0 (4.0, 15.5)	0.263	10.0 (5.0, 20.0)	12.0 (10.0, 20.0)	0.209
EHRA, *n* (%)			<0.001			0.353			0.399
I	2 (1.9%)	35 (8.2)		2 (2.4)	0		1 (1.1)	1 (6.7)	
II	49 (45.8)	252 (58.9)		39 (47.6)	10 (40.0)		41 (44.6)	8 (53.3)	
III	56 (52.3)	136 (31.8)		41 (50.0)	15 (60.0)		50 (54.3)	6 (40.0)	
IV	0	2 (0.4)		0	0		0	0	
Stroke, *n* (%)	8 (7.5)	34 (7.9)	0.872	4 (4.9)	4 (16.0)	0.064	8 (8.7)	0	0.286
HF, *n* (%)	10 (9.3)	44 (10.3)	0.774	8 (9.8)	2 (8.0)	0.792	10 (10.9)	0	0.205
HTN, *n* (%)	56 (52.3)	247 (57.7)	0.316	45 (54.9)	11 (44.0)	0.340	48 (52.2)	81 (53.3)	0.934
CAD, *n* (%)	23 (21.5)	95 (22.2)	0.876	20 (24.4)	3 (12.0)	0.148	16 (17.4)	7 (46.7)	0.010
PAD, *n* (%)	6 (5.6)	34 (7.9)	0.411	5 (6.1)	1 (4.0)	0.571	6 (6.5)	0	0.395
Hyperlipidemia, *n* (%)	42 (39.6)	166 (38.8)	0.874	32 (39.5)	10 (40.0)	0.965	35 (38.0)	7 (46.7)	0.394
CKD, *n* (%)	6 (5.6)	34 (7.9)	0.411	5 (6.1)	1 (4.0)	0.571	5 (5.4)	1 (6.7)	0.605
DM, *n* (%)	18 (16.8)	81 (18.9)	0.616	14 (17.1)	4 (16.0)	0.585	16 (17.4)	2 (13.3)	0.518
Corticosteroid, *n* (%)	17 (15.9%)	–	–	16 (19.5%)	1 (4.0%)	0.122	11 (12.0%)	6 (40.0%)	0.017
DMARD, *n* (%)	21 (19.6%)	–	–	21 (25.6%)	0	0.011	20 (21.7%)	1 (6.7%)	0.311
LAD, mm	38.8 ± 5.2	39.7 ± 5.6	0.126	38.8 ± 5.0	38.9 ± 5.8	0.939	38.7 ± 5.3	39.5 ± 4.4	0.620
LVEF, %	63.1 ± 6.0	63.5 ± 7.6	0.610	63.2 ± 6.0	62.8 ± 6.1	0.796	63.2 ± 5.8	62.4 ± 7.5	0.643
sPAP, mmHg	32.6 ± 7.3	30.3 ± 7.5	0.011	33.3 ± 7.2	30.3 ± 7.5	0.075	32.6 ± 7.4	32.5 ± 6.4	0.975
mPAP, mmHg	22.0 ± 4.8	20.5 ± 5.1	0.009	22.5 ± 4.7	20.5 ± 4.7	0.075	22.0 ± 4.9	22.0 ± 4.2	0.975
Median follow‐up, months (IQR)	27.9 (8.1, 55.0)	39.2 (12.0, 61.6)	0.088	23.8 (6.0, 57.5)	35.3 (16.4, 47.9)	0.667	25.7 (8.3, 53.4)	48.9 (7.0, 71.2)	0.339

Abbreviations: AD, autoimmune disease; AF, atrial fibrillation; CAD, coronary artery disease; CKD, chronic kidney disease; DM, diabetes mellitus; DMARD, disease‐modifying antirheumatic drugs; EHRA, European Heart Rhythm Association symptom classification; HF, heart failure; HTN, hypertension; IQR, interquartile range; LAD, left atrium diameter; LVEF, left ventricular ejection fraction; mPAP, mean pulmonary artery pressure; *P*1, *p* value between AD group and non‐AD group; *P*2, *p* value between connective tissue diseases group and organ‐specific diseases; *P*3, *p* value between single AD group and multiple AD group; PAD, peripheral artery disease; sPAP, systolic pulmonary artery pressure.

In patients with AD, 17 (15.9%) patients were under regular oral corticosteroid therapy, and 21 (19.6%) patients with CTD were on regular DMARD therapy (4 methotrexate, 5 sulfasalazine, 8 leflunomide, 3 hydroxychloroquine). Patients with multiple ADs had a higher proportion of corticosteroid usage (40.0% vs. 12.0%, *p* = 0.017).

Patients with AD had significantly higher levels of hs‐CRP (2.16 [0.82, 5.33] vs. 0.98 [0.47, 1.70] mg/L; *p* < 0.001). No disparities in levels of hs‐CRP were detected between subgroups of AD patients, and no significant differences existed in levels of blood cell count and proportions between patients with and without AD, as well as in subgroups of AD patients (Supporting Information: Table 1).

### 3.2 CA

PVI was achieved in every involved patient. Linear ablation and CFAE ablation were conducted in a comparable proportion of patients in the AD group and the non‐AD group (Table 2). Notably, non‐PV triggers were detected and intervened in a significantly higher percentage of patients in the AD group (18.7% vs. 8.4%, *p* = 0.002). Among all non‐PV triggers, SVC presented as the most common origin (in 33 non‐AD patients and 19 AD patients). Other trigger origins included left atrial appendage in three patients, right atrial appendage in two patients, tricuspid valve annulus in one patient, and cristae terminalis in one patient. Beyond routine linear ablation at MI, CTI, and RL, additional linear ablation was conducted in 15 patients (11 [10.3%] AD patients and 5 [1.2%] non‐AD patients, *p* < 0.001), including posterior wall isolation in 8 patients (4 AD and 4 non‐AD), septal linear ablation in 6 patients (5 AD and 1 non‐AD), and anterior wall line in 4 patients (3 AD and 1 non‐AD). No significant disparities in ablation strategy were detected in AD subgroups.

**Table 2:** Ablation strategy in the index and redo procedures.

Ablatio target (*n*, %)	AD (*n* = 107)	Non‐AD (*n* = 428)	*P*1	Connective tissue diseases (*n* = 82)	Organ‐specific diseases (*n* = 25)	*P*2	Single AD (*n* = 92)	Multiple AD (*n* = 15)	*P*3
*Index procedure*									
MI	47 (43.9%)	189 (44.1%)	0.973	38 (46.3%)	9 (36.0%)	0.337	39 (42.3%)	8 (53.3%)	0.449
CTI	64 (59.8%)	255 (59.5%)	0.965	50 (61.0%)	14 (56.0%)	0.657	54 (58.7%)	10 (66.7%)	0.559
RL	50 (46.7%)	181 (42.3%)	0.407	37 (45.1%)	13 (52.0%)	0.546	42 (45.6%)	8 (53.3%)	0.580
Any linear ablation	69 (64.5%)	267 (62.4%)	0.687	54 (65.8%)	15 (60.0%)	0.592	57 (61.9%)	12 (80.0%)	0.176
CFAE	10 (9.3%)	21 (4.9%)	0.074	8 (9.7%)	2 (8.0%)	1.000	7 (7.6%)	3 (20.0%)	0.247
Non‐PV trigger	20 (18.7%)	36 (8.4%)	0.002	18 (21.9%)	2 (8.0%)	0.203	17 (18.5%)	3 (20.0%)	1.000
Cardioversion	34 (31.8%)	137 (32.0%)	0.990	26 (31.7%)	8 (32.0%)	0.993	30 (32.6%)	4 (26.7%)	0.853
Recur	44(41.1%)	155(36.2%)	0.210	38 (46.3%)	6 (24.0%)	0.084	39 (42.4%)	5 (33.3%)	0.450
Redo	35 (79.5%)	40 (25.8%)	<0.001	28 (34.1%)	6 (100.0%)	0.191	33 (84.6%)	2 (40.0%)	0.050
PAF redo	23 (65.7%)	38 (95.0%)	0.001	20 (71.4%)	3 (50.0%)	0.163	21 (63.6%)	2(100%)	0.425
*Redo procedure*									
PV gaps	29 (82.8%)	38 (95.0%)	0.185	24 (85.7%)	5 (87.3%)	0.647	28 (84.8%)	1 (50.0%)	0.318
Linear ablation	27 (77.1%)	22 (55.0%)	0.027	20 (71.4%)	6 (100%)	0.171	26 (78.8%)	1 (50.0%)	0.410
Non‐PV trigger	9 (25.7%)	5 (12.5%)	0.143	6 (7.3%)	3 (50.0%)	0.174	9 (27.3%)	0	–
CFAE	4 (11.4%)	3 (7.5%)	0.853	4 (14.3%)	0	–	3 (9.1%)	1 (50.0%)	0.218

Abbreviations: AD, autoimmune disease; CFAE, complex fractionated atrial electrograms; CTI, cavotricuspid isthmus; MI, mitral isthmus; *P*1, *p* value between AD group and non‐AD group; *P*2, *p* value between connective tissue diseases group and organ‐specific diseases; *P*3, *p* value between single AD group and multiple AD group; PAF, paroxysmal atrial fibrillation; PV, pulmonary vein; RL, roofline.

The incidence of procedure‐related complications was relatively low in both groups, including two cases of cardiac tamponade (one in a 75‐year‐old female in the non‐AD group and one in a 78‐year‐old female with RA and Grave disease), one case of postcardiac injury syndrome in a 68‐year‐old female with RA. Other mild complications included five cases of pseudoaneurysm and three cases of arteriovenous fistula. No periprocedural thromboembolism event occurred in either group.

### 3.3 Procedure outcomes

**Figure 1:**
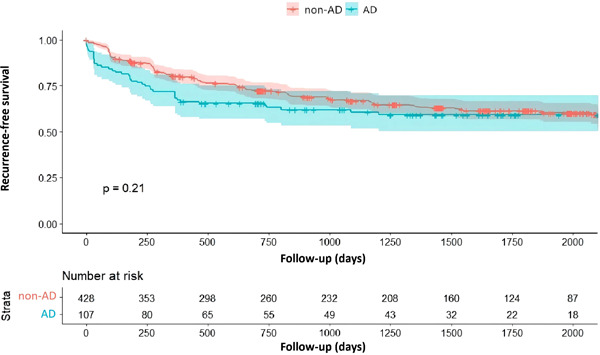
Kaplan–Meier curve for AF recurrence after index catheter ablation for AF in AD and non‐AD patients. AD, autoimmune disease; AF, atrial fibrillation.

**Figure 2:**
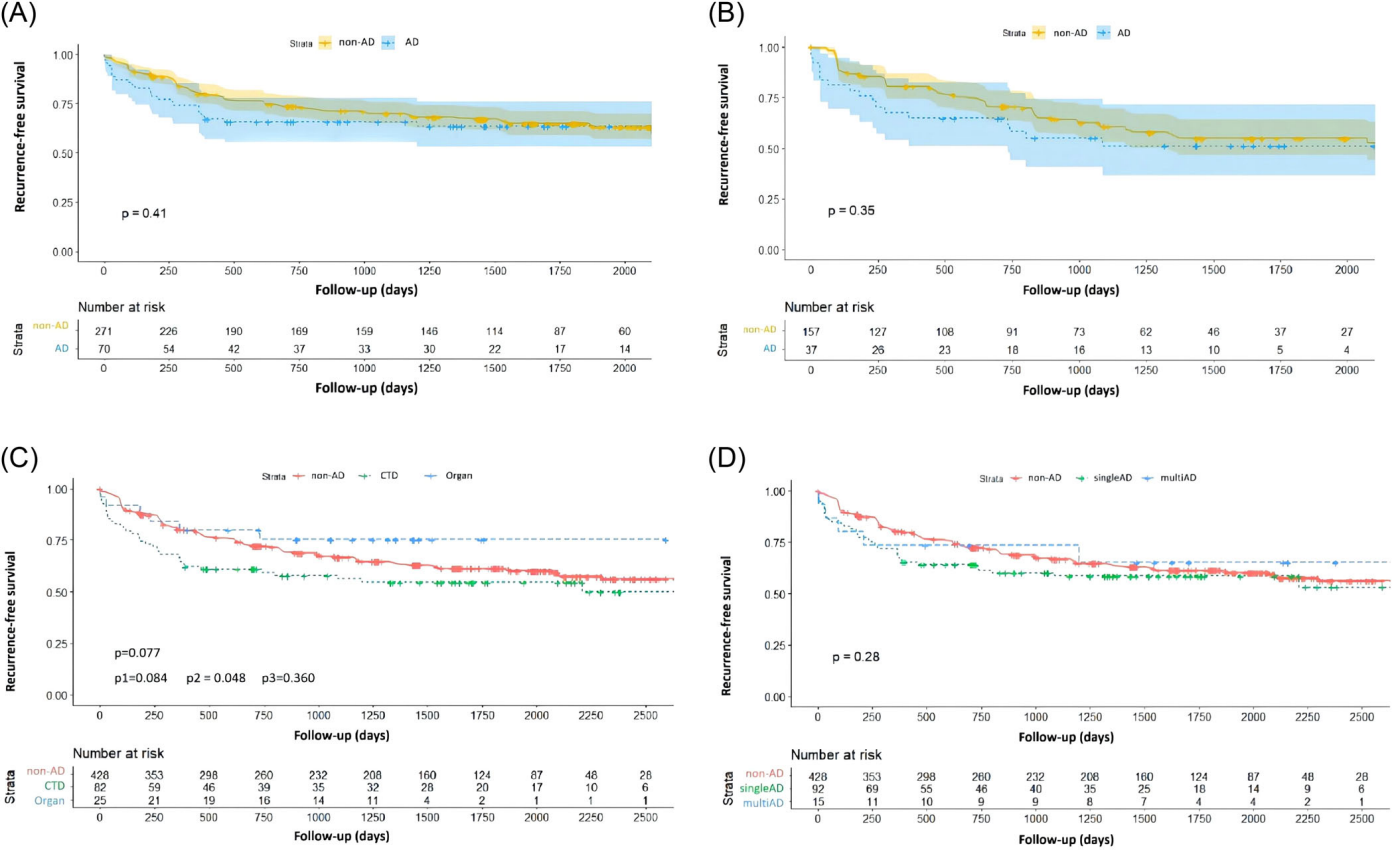
Kaplan‐Meier curve for atrial fibrillation (AF) recurrence after index catheter ablation for AF in subgroups. (A) In patients with paroxysmal AF; (B) in patients with persistent AF; (C) in patients with connective tissue disease (CTD) and organ‐specific AD; and (D) in patients with single and multiple AD. AD, autoimmune disease; p1, patients with CTD compared to patients with organ‐specific AD; p2, patients with CTD compared to non‐AD patients; p3, patients with organ‐specific AD compared to non‐AD patients.

During a median follow‐up duration of 36.3 (11.8–60.5) months, the recurrence rate was comparable in the two groups (AD group, *n* = 44, 41.1%, non‐AD group, *n* = 155, 36.2%, *p* = 0.21, HR: 1.23, 95% CI: 0.86–1.76, Figure 1). Further comparison of recurrence risk between patients with and without AD in the PAF and the PsAF subgroups also revealed no significant differences (Figure 2A,B). Notably, patients with AD tended to experience more early AF recurrence (36.4% vs. 13.5%, *p* = 0.001) and less very late recurrence (25.0% vs. 46.4%, *p* = 0.011, Supporting Information: Table 2).

In AD subgroups, patients with organ‐specific AD presented with a lower risk of recurrence compared to patients with CTD but was not of statistical significance (24.0% vs. 46.3%, *p* = 0.084, HR: 0.48, 95% CI: 0.20–1.13). Compared to non‐AD patients, patients with CTD were at a higher risk of recurrence (46.3% vs. 36.2%, *p* = 0.049, HR: 1.43, 95% CI: 1.01–2.05) (Figure 2C). The risk of recurrence was comparable in patients with single and multiple AD (Figure 2D).

### 3.4 Redo ablation procedure

Forty patients in the non‐AD group and 35 patients in the AD group received redo procedures.

A significantly higher proportion of patients in the AD group received a redo procedure (35 in 44 [79.5%] vs. 40 in 155 [25.8%], *p* < 0.001). No significant disparities in baseline characteristics were detected between patients receiving and not receiving a redo procedure (Supporting Information: Table 3). Redo procedures for PAF accounted for 65.7% of the AD group and 95.0% in the non‐AD group (*p* = 0.001). The most common target in redo procedures was recovered PV conduction (82.8% in the AD group and 95.0% in the non‐AD group, *p* = 0.185). De novo or touch‐up linear ablation was conducted in a higher percentage of patients with AD (77.1% vs. 55.0%, *p* = 0.027).

### 3.5 Predictors of risk of recurrence after CA procedure

Based on previous studies and expected clinical relevance, the following variables were entered into a Cox logistic regression model: age, gender, AF type, duration of AF history, history of HF, HTN, CAD, CKD, DM, regular usage of oral corticosteroid, DMARD, LAD, LVEF, and hs‐CRP.

Univariate analysis in AD patients revealed that AF history (HR: 1.11, 95% CI: 1.00–1.20, *p* < .001), history of HF (HR: 2.40, 95% CI: 1.10–5.40, *p* = 0.037), and CKD (HR: 3.10, 95% CI: 1.20–8.00, *p* = 0.017) were predictors of AF recurrence. In the multivariate analysis, duration of AF history (HR: 1.11, 95% CI: 1.06–1.18, *p* < 0.001) and regular usage of corticosteroids (HR: 2.42, 95% CI: 1.12–5.24, *p* = 0.025) were independent predictors of AF recurrence (Table 3). In the non‐AD group, the female gender (HR: 1.50, 95% CI: 1.10–2.07, *p* = 0.012), duration of AF history (HR: 1.00, 95% CI 1.00–1.10, *p* = 0.018), HF (HR: 1.90, 95% CI: 1.20–2.90, *p* = 0.003), LAD (HR: 1.00, 95% CI: 1.00–1.10, *p* = 0.007) were predictors of AF recurrence, while HTN presented as a protective factor (HR: 0.70, 95% CI: 0.51–0.96, *p* = 0.003). Multivariate analysis found female gender (HR: 1.51, 95% CI: 1.10–2.07, *p* = 0.012), AF history (HR: 1.03, 95% CI 1.00–1.05, *p* = 0.034), and LAD (HR: 1.03, 95% CI: 1.00–1.06, *p* = 0.030) were independent predictors of AF recurrence.

**Table 3:** Univariable and multivariable logistic regression analysis for predictors of recurrence.

	HR represents	AD group				Non‐AD group			
		Univariate model HR (95% CI)	*p* Value	Multivariate model HR (95% CI)	*p* Value	Univariate model HR (95% CI)	*p* Value	Multivariate model HR (95% CI)	*p* Value
Age	Per year increase	1.00 (0.97–1.00)	0.900	–	–	1.00 (0.99–1.00)	0.260	–	–
Gender	Female versus male	0.80 (0.44–1.50)	0.460	–	–	**1.50 (1.1–2.10)**	**0.012**	**1.51 (1.10–2.07)**	**0.012**
AF type	PsAF versus PAF	1.30 (0.71–2.40)	0.390	–	–	1.30 (0.92–1.70)	0.160	–	–
AF history	Per year increase	**1.10 (1.00–1.20)**	**<0.001**	**1.11 (1.06–1.18)**	**<0.001**	**1.00 (1.00–1.10)**	**0.018**	**1.03 (1.00–1.05)**	**0.034**
HF	Yes versus no	**2.40 (1.10–5.40)**	**0.037**	–	–	**1.90 (1.20–2.90)**	**0.003**	–	–
HTN	Yes versus no	1.20 (0.68–2.30)	0.470	–	–	**0.70 (0.51–0.96)**	**0.003**	–	–
CAD	Yes versus no	1.00 (0.50–2.20)	0.530	–	–	0.72 (0.48–1.10)	0.120	–	–
CKD	Yes versus no	**3.10 (1.20–8.00)**	**0.017**	–	–	0.72 (0.37–1.4)	0.350	–	–
DM	Yes versus no	0.81 (0.34–1.90)	0.620	–	–	0.86 (0.57–1.3)	0.500	–	–
LAD	Per mm increase	1.10 (1.00–1.10)	0.068	–	–	**1.00 (1.00–1.10)**	**0.007**	**1.03 (1.00–1.06)**	**0.030**
LVEF	Per percent increase	0.96 (0.92–1.00)	0.088	–	–	0.97 (0.95–1.14)	0.150	–	–
Corticosteroid	Yes versus no	2.00 (0.97–4.00)	0.061	**2.42 (1.12–5.24)**	**0.025**	–	–	–	–
DMARD	Yes versus no	1.00 (0.49–2.10)	0.950	–	–	–	–	–	–
hs‐CRP	Per mg/L increase	0.99 (0.95–1.00)	0.630	–	–	1.06 (0.85–1.33)	0.580	–	–

*Note*: Bold values indicate *p* < 0.05.

Abbreviations: AD, autoimmune disease; AF, atrial fibrillation; CAD, coronary artery disease; CI, confidence interval; CKD, chronic kidney disease; DM, diabetes mellitus; DMARD, disease‐modifying antirheumatic drugs; HF, heart failure; HR, hazard ratio; hs‐CRP: hypersensitive C‐reactive protein; HTN, hypertension; LAD, left atrial diameter; LVEF, left ventricular ejection fraction; PAF, paroxysmal atrial fibrillation.

## 4 DISCUSSION

Despite the small sample size and retrospective nature, to our knowledge, our study represents the largest observational cohort of patients with AD undergoing CA procedures for AF, which covered a larger spectrum of ADs compared with previous research.^15^, ^16^ Major findings of this research include:

(1) Patients with AD tended to suffer from more severe AF‐related symptoms;
(2) ablation procedures in patients with AD were likely to be more sophisticated than those in non‐AD patients, which required more interventions at non‐PV triggers and extra linear ablation (e.g., box isolation, septal line, and anterior line);
(3) during a long‐term follow‐up, patients with AD did not present a higher risk of atrial arrhythmia recurrence compared to non‐AD patients after a single procedure. However, there is an increased risk of postablation recurrence in patients with CTD. Compared to non‐AD patients, AD patients experienced more early recurrence; and
(4) the duration of AF history is an independent predictor of AF recurrence in both groups, and regular usage of corticosteroids was associated with AF recurrence in patients with AD. The level of hs‐CRP failed to present any predictive value of AF recurrence in both groups.

Evidence on the treatment of AF in AD patients is extremely scant. Wen et al. reported that the success rate of CA for AF in patients with RA was comparable to that of patients without RA.^15^ However, a recent study came out with exactly the opposite conclusion: patients with RA were at higher risk of AF recurrence and receiving repeated ablation.^16^ Our research revealed that, with a similar profile of cardiovascular risk factors, AD patients were not at an increased risk of recurrence during the long‐term follow‐up. However, considering the negative influence of inflammation status, patients with AD are expected to have more complicated LA substrates. In our study, although LA substrate was not routinely mapped, a higher prevalence of non‐PV triggers in patients with AD and a more frequent nonroutine linear ablation in the index procedure could potentially corroborate this hypothesis.

In our study population, the preablation hs‐CRP was higher in the AD group compared to the non‐AD group but was not of predictive value for recurrence like the previously reported.^17^, ^18^ The reason might be explained by the difference in the level of hs‐CRP between the AD group and the non‐AD group was not clinically prominent although statistically significant, which may reflect that some patients in the AD group were not in active disease status. However, some results from our study still indicated that an overall inflammatory status could negatively influence the efficacy of the CA procedure, as patients with CTD, which generally suffered from a systemic inflammatory status,^1^ had a higher risk of arrhythmia recurrence compared to the patients with organ‐specific AD (with a more localized inflammation status). In addition, the multivariate analysis revealed that regular oral corticosteroid therapy at the time of ablation was an independent predictor of arrhythmia recurrence in the AD group, which may indicate that an active disease status (as reflected by demanding corticosteroid therapy), can exert negative effects on the efficacy of ablation procedure, even off‐set the expected protective effects on postablation recurrence by corticosteroid therapy.^19^ In addition, long‐term treatment with corticosteroids per se is also likely to contribute to high cardiovascular risk,^20^, ^21^ incurring an elevated risk of AF recurrence.

Another problem with patients with AD is that the LA function could already be impaired by an inflammatory status. Therefore, it is reasonable to caution against further impairment of atrial function by ablation energy.^22^ In our study, the preablation sPAP and mPAP were slightly higher in the AD group than that in the non‐AD group. We speculated that an inflammation status combined with a further increase in LA pressure contributed to the higher risk of early recurrence. Further prospective studies are needed to confirm and explore the mechanism of a more frequent early recurrence and establish the relationship between the degree of inflammation status, and impairment of atrial function with adverse outcomes in CA for AF in patients with AD.

### 4.1 Limitations

Our study has several inherent limitations. First, the limited case number precluded further analysis of the impact of an individual type of AD on procedural outcomes. Considering the component of the AD group in our study, patients with RA and AS were better represented, while the impact of rarer AD‐like SLE, systemic sclerosis on the outcome of AF ablation is still worth further investigation as only sparse patients with these diseases were involved in our study. In addition, levels of biomarkers reflecting the disease activity were lacking in our baseline data, making us unable to establish the correlation between the disease activity of AD and the procedure outcome. Moreover, potential ascertainment bias may exist as patients with ADs generally have more opportunities to get contact with the health care system and increased chances of AF confirmation after AF ablation.

## CONFLICTS OF INTEREST STATEMENT

Dr. Chang‐Sheng Ma has received honoraria for presentations from AstraZeneca, Bayer Healthcare, Boehringer Ingelheim, Bristol‐Myers Squibb, Johnson & Johnson, and Pfizer. Dr. De‐Yong Long and Dr. Jian‐Zeng Dong have received honoraria for presentations from Johnson & Johnson and Abbott. The remaining authors declare no conflict of interest.

## Supporting information

Supporting information.

## Data Availability

The datasets generated and/or analyzed during the current study are not publicly available due to the protection of patient privacy but are available from the corresponding author upon reasonable request.
