# Supplementary material for: Catheter ablation of atrial fibrillation in patients with autoimmune disease: A propensity score matching study based on the China Atrial Fibrillation Registry
Source: Clin Cardiol. 2023 May 22;46(7):801–9. doi: 10.1002/clc.24036 (PMC10352975; doi:10.1002/clc.24036)
Supplement: Supplementary file 1 — Supporting information. [file CLC-46-801-s001.docx]

**Supplementary Table 1** Hematological index.

| **Variable** | **AD**  **(n=107)** | **non-AD**  **(n=428)** | **P1** | **Connective**  **tissue diseases**  **(n=82)** | **Organ-specific**  **Diseases (n=25)** | **P2** | **Single  AD**  **(n=92)** | **Multiple AD**  **(n=15)** | **P3** |
| --- | --- | --- | --- | --- | --- | --- | --- | --- | --- |
| **hs-CRP**  **(mg/L, IQR)** | 2.16  (0.82, 5.33) | 0.98  (0.47, 1.70) | ＜0.001 | 2.36  (0.89, 5.56) | 1.11  (0.68, 3.89) | 0.223 | 2.07  (0.82, 5.20) | 3.58  (0.70, 7.31) | 0.641 |
| **WBC (*10^9)** | 6.86±1.98 | 6.62±1.61 | 0.422 | 6.79±1.93 | 7.03±2.00 | 0.598 | 6.86±1.94 | 6.86±2.24 | 0.988 |
| **Lym(%)** | 28.20±11.01 | 30.06±8.19 | 0.271 | 29.37±13.01 | 27.50±8.02 | 0.547 | 11.61±1.42 | 6.59±1.99 | 0.786 |
| **Mono(%)** | 6.74±7.86 | 5.57±1.91 | 0.255 | 7.33±9.88 | 5.47±1.21 | 0.352 | 6.81±8.44 | 6.30±2.33 | 0.817 |
| **Ne(%)** | 61.29±12.21 | 61.36±9.41 | 0.967 | 60.37±14.00 | 63.80±9.08 | 0.259 | 61.33±12.85 | 61.05±7.63 | 0.935 |
| **Lym(*10^9)** | 1.93±0.77 | 2.45±3.76 | 0.173 | 1.90±0.79 | 1.95±0.76 | 0.824 | 1.92±0.78 | 2.01±0.73 | 0.651 |
| **Mono(*10^9)** | 0.54±1.07 | 0.36±0.13 | 0.185 | 0.63±1.35 | 0.39±0.15 | 0.381 | 0.56±1.15 | 0.44±0.24 | 0.677 |
| **Ne(*10^9)** | 4.30±1.63 | 4.13±1.47 | 0.502 | 4.22±1.71 | 4.52±1.52 | 0.447 | 4.31±1.65 | 4.24±1.56 | 0.882 |

AD: autoimmune disease; hs-CRP: hypersensitive C reactive protein; WBC: white blood cell; Lym: lymphocyte; Mono: monocyte; Ne: neutrophils; P1: AD group vs. non-AD group; P2: connective tissue disease group vs. organ-specific disease group; P3: patients with multiple Ads vs. patients with single AD

**Supplementary Table 2** Recurrence distribution in AD and non-AD groups

|  | **AD group** | **non-AD group** | **P** |
| --- | --- | --- | --- |
| **Early recurrence** | 16 (36.4%) | 21 (13.5%) | 0.001 |
| **Late recurrence** | 17 (38.6%) | 62 (40.0%) | 0.507 |
| **Very late recurrence** | 11 (25.0%) | 72 (46.4%) | 0.011 |

AD: autoimmune disease

**Supplementary Table 3** Characteristics of patients who experienced recurrence grouped by redo and non-redo choices

|  | **redo (n=75)** | **no-redo (n=124)** | **P value** |
| --- | --- | --- | --- |
| **Age (y)** | 65.2±10.9 | 65.1±10.9 | 0.86 |
| **Female (n,%)** | 34 (45.3) | 64 (51.6) | 0.48 |
| **AF duration(y)** | 3.32 (1.04, 7.09) | 3.80 (2.00,5.90) | 0.51 |
| **LAD (mm)** | 40.9±5.5 | 39.1±5.3 | 0.12 |
| **LVEF (%)** | 63.3±7.4 | 64.0±6.6 | 0.59 |
| **PeAF (n,%)** | 13 (17.3%) | 60 (48.4%) | 0.58 |
| **Stroke (n,%)** | 6 (8%) | 10 (8.1%) | 0.68 |
| **HF (n,%)** | 9 (12%) | 26 (21.0%) | 0.69 |
| **HTN (n,%)** | 44 (58.7%) | 57 (46.0%) | 0.08 |
| **CAD (n,%)** | 16 (21.3%) | 24 (19.4%) | 0.82 |
| **DM (n,%)** | 10 (13.3%) | 20 (16.1%) | 0.99 |
| **CKD (n,%)** | 6 (8%) | 10 (8.1%) | 1.00 |
| **Hyperlipid (n,%)** | 26 (34.7%) | 46 (37.1%) | 0.09 |

LAD: left atrial diameter; LVEF: left ventricular ejection fraction; PeAF: persistent atrial fibrillation; HF: heart failure; HTN: hypertension; CAD: coronary artery disease; DM: diabetes mellitus; CKD: chronic kidney disease
